# PP14 Recombinant Human Erythropoietin For Sickle Cell Disease And Brazilian Healthcare System Sustainability: Cost-Effectiveness And Budget Impact Analysis

**DOI:** 10.1017/S0266462324001879

**Published:** 2025-01-07

**Authors:** Ludmila Gargano, Clarisse Lobo, Haliton Alves De Oliveira, Rosa Lucchetta

## Abstract

**Introduction:**

Sickle cell disease (SCD) is a rare disease, including renal complications. Erythropoiesis-stimulating agents (i.e., recombinant human erythropoietin, rHuEPO) are recommended for SCD and renal impairment. Evidence suggests its effectiveness in raising hemoglobin (Hb), which may reduce the need for regular blood transfusions. Cost-effectiveness analysis (CEA) and budget impact analysis (BIA) of rHuEPO were performed from the Brazilian Unified Health System (SUS) perspective.

**Methods:**

A decision tree was created to assess rHuEPO’s impact on reducing blood transfusions. Quality-adjusted life year (QALY) for transfusion dependency and direct medical costs related to rHuEPO and transfusion were considered. SCD patients with worsening kidney function and Hb levels enter the model with an indication of receiving regular transfusions; those who proceed to the rHuEPO+standard care arm are likely to have clinically relevant elevation in Hb levels (i.e., <1.5 g/dL), suspending transfusions, and those who do not respond to treatment continue to receive regular transfusions. BIA population was estimated based on epidemiological data, considering direct costs over a five-year horizon.

**Results:**

rHuEPO+standard care compared to standard care generated 36.8 percent less need for transfusions, resulting in an increase of 0.033 QALY and a saving of BRL11,564 (USD2,362) per patient/year. rHuEPO was the dominant alternative; that is, there was greater clinical benefit and lower total cost. At BIA, the eligible population was 5,274 patients per year, on average. The direct cost of acquiring rHuEPO for the total eligible population summed BRL13,737,129 (USD2,806,016) in five years. However, considering the estimated effectiveness of CEA in reducing transfusions, BIA demonstrated savings of BRL96,545,791 (USD19,720,936) accumulated over five years.

**Conclusions:**

Health technology assessments showing the new alternative as dominant are uncommon, especially in rare diseases, where patented orphan drugs usually have increased costs, which is not the case of rHuEPO. In this analysis, rHuEPO remained the dominant alternative and its incorporation would result in savings that may contribute to the sustainability of the Sistema Único de Saúde, Brazil’s national health system.

